# Mass Spectrometric Analysis of Bisphenol A Desorption from Titania Nanoparticles: Ammonium Acetate, Fluoride, Formate, and Hydroxide as Chemical Desorption Agents

**DOI:** 10.3390/mps1030026

**Published:** 2018-07-19

**Authors:** Seyed Mohammad Majedi, Edward P. C. Lai

**Affiliations:** Department of Chemistry, Ottawa-Carleton Chemistry Institute, Carleton University, Ottawa, ON K1S 5B6, Canada; mohammad.majedi@carleton.ca

**Keywords:** adsorption, bisphenol A, desorption, direct infusion, mass spectrometry, nanoparticles, titania

## Abstract

Bisphenol A (BPA) is a widely used chemical in several consumer products and a well-studied environmental toxicant, and therefore, its accurate measurement is highly demanded. However, the co-presence of nanoparticles as an emerging class of contaminants could result in inaccurate determination of BPA due to binding of BPA onto nanoparticle surface. In this study, mass spectrometry (MS) was used to investigate desorption of BPA bound on the surface of titania (TiO_2_) nanoparticles in water. Ammonium acetate, fluoride, formate, and hydroxide were evaluated as chemical agents for their desorption capabilities. The percentages of recovery, adsorption, and desorption were determined by this new method without requiring any prior separation of nanoparticles from BPA. MS analysis demonstrated the desorption of BPA by 10–20 mM of ammonium hydroxide for a mixture of 5 µg/mL BPA and 10 µg/mL TiO_2_ nanoparticles, with a desorption efficiency of 72 ± 1%. Due to adsorption of BPA onto the nanoparticle surface that was inefficient for electrospray ionization, the resulting abundance of target ions could be reduced in the detection of BPA by mass spectrometry. As such, these findings collectively promise an accurate determination of the total BPA concentration in water whether it exists in the free or bound form. Efficient desorption of contaminants from the surface of nanoparticles would improve the accuracy of the contaminant analysis by mass spectrometry.

## 1. Introduction

Bisphenol A (BPA) is a high production volume compound for use as a monomer to make epoxy resins and polycarbonate plastics for various consumer product applications (including food-contact materials). It is a widely studied endocrine disrupting chemical and a pervasive environmental toxicant with known reproductive effects on sperm parameters and hormone levels [1]. This micro-contaminant can affect exposed organisms through multiple modes of action—estrogenic, androgenic, and anti-androgenic [2]. For instance, exposure to BPA at environmentally relevant concentrations impairs germ cell development in first trimester human fetal testis [3]. The primary route of exposure to BPA in the general population is through oral intake, but it is impossible to moderate BPA exposure by diet in a real-world setting [4]. Replacement of BPA with bisphenol F, P and S has been considered as a solution but these alternatives also produce toxic by-products [5].

In the European Union, the law severely restricted BPA use due to its endocrine disrupting properties. On 12 February 2018, European regulation 213 has amended EU regulation 10/2011 on the level of BPA in plastic food contact materials. The specific migration limit (SML) of these materials reduced from 0.6 to 0.05 mg BPA/kg. The amendment should be applicable on 6 September 2018. The concentrations of BPA in sewage influent and effluent samples from Beijing, China were determined to be 636–1200 ng/L by an isotope-dilution ultra-performance liquid chromatography–electrospray tandem mass spectrometry method (for product ions of 695 > 171 and 699 > 171) combined with dansylation [6]. Chemicals showing structural or functional similarity to BPA, commonly called BPA analogues, have recently drawn scientific attention due to their common industrial and commercial application as a substitute for BPA. Increasingly bisphenol AF, bisphenol M, bisphenol P, and bisphenol S are considered a good replacement for BPA. Unfortunately, it seems that all BPA analogues show comparable biological activity, including hormonal disruption, toxicity and genotoxicity [7]. Several bisphenols existed in ready-to-eat plastic packaged foods consumed by preschoolers, with bisphenol S detected in high concentration [8]. A comprehensive review has recently been published of the various techniques employed for measuring bisphenols and their derivatives in different biological samples and consumer products by mass spectrometry, ultraviolet, and fluorescence detection coupled with liquid and gas chromatographic separation techniques. Among all detection techniques, mass spectrometry showed the highest accuracies, the best selectivity, and the lowest limits of detection [9]. While many remediation technologies can successfully be applied for the removal of BPA from any source of contaminated water [10,11,12,13,14,15], it is more critical to consider the existence of engineered nanoparticles such as titania (TiO_2_) that binds BPA inadvertently to alter their bioaccessibility and bioavailability [16]. Particularly, there is mounting evidence that sorption of BPA to microplastic particles led to a reduction of BPA in the aqueous phase and the particles loaded with BPA were ingested by freshwater zooplankton [17]. TiO_2_ possesses antibacterial and photocatalytic properties for coating a variety of building, food packaging, and water treatment materials [18,19,20]. TiO_2_ polymorphs (anatase, rutile, and brookite) and nanocomposite mixtures are suitable for the catalytic wet air oxidation of BPA [21]. TiO_2_/bismuth(III) oxide (Bi_2_O_3_)composite as an effective visible light photocatalyst was applied for the degradation of aqueous bisphenol A solutions [22]. BPA has two phenolic functional groups that can bind with the surface of (and hence be adsorbed by) TiO_2_ nanoparticles via specific interactions [23]. Apparently, TiO_2_ nanoparticles in combination with BPA could increase BPA bioavailability and uptake into cells and organisms [16]. The adsorption of BPA onto TiO_2_ nanoparticles even at low concentration levels was previously reported with no observed differences in the adsorption capacities among various cell cultures and therefore in their toxicological effects [24]. Such correlational studies have been recently reviewed to link between the co-presence of nanoparticles with contaminants and their synergistic effects on bioaccessibility and joint toxicity to living organisms [25]. Whether it is the fluid environment of a target organism or the hydrophobic lipid bilayer of gastrointestinal cell membranes, bioavailability usually requires a preliminary desorption of BPA from the surface of nanoparticles. However, desorbed species may adopt a very different molecular structure than that for BPA, thereby making it very difficult for environmental or food toxicologists to predict its ultimate estrogenic activity without being cytotoxic. Indeed, the identification of by-products arising from adsorption treatments is of primary importance because desorbed compounds can be more hazardous than their precursors [26]. Fortunately, mass spectrometry (MS) can be used to detect desorbed species, possibly together with a proposal of plausible desorption mechanism or pathways involved.

In the present work, BPA was chosen for use as a representative prototype of endocrine disrupting chemicals. Herein, the adsorption/degradation of BPA in water onto/by TiO_2_ nanoparticles was investigated by using direct-infusion MS, without liquid chromatography (LC). Direct-infusion MS enabled us to analyze BPA in the presence of nanoparticles without requiring the separation of nanoparticles prior to analysis. It further enhanced the sensitivity and selectivity to target the adsorbed/desorbed BPA molecules, and demonstrated the interferences of solid phase (here, nanoparticles) with electrospray ionization. We aimed to evaluate desorption capabilities of four chemical agents, ammonium acetate (NH_4_AC), fluoride (NH_4_F), formate (NH_4_COOH), and hydroxide (NH_4_OH), and to identify the by-products formed under acid-base, complexometric, oxidative, and photocatalytic desorption conditions. However, this study was not focused on the photocatalytic degradation of BPA since such a process had previously been well studied and reported [22,26]. It was understood that if the polarity was tuned by adding solvents such as acetonitrile or isopropanol into a water sample, BPA could be desorbed but the dilution factor (e.g., for 1:1 *v*/*v* of the solvent:water) might not be acceptable in trace analysis. As mentioned earlier, while the present systematic study of adsorption/desorption of BPA onto/from TiO_2_ nanoparticles demonstrated the applicability of direct-infusion MS for the determination of BPA with enhanced accuracy, detection of trace levels of BPA may require various sample preparation techniques for preconcentration of the analyte and minimization of any matrix effect. However, the adsorption of BPA to the nanoparticle surface could prevent high recovery of BPA in the bound form. It necessitates desorption of BPA using the chemical agents developed in this study prior to preconcentration of trace BPA levels.

## 2. Materials and Methods

### 2.1. Reagents and Chemicals

All chemicals were obtained from commercial sources and were used as received. Bisphenol A (≥99% C_15_H_16_O_2_, molecular weight (M.W.) = 228.29), methanol (LC-MS grade), and TiO_2_ nanopowder (99% anatase, <25 nm particle size) were purchased from Sigma-Aldrich (Oakville, ON, Canada). Ultrapure water, from a Milli-Q system (Millipore, Milford, MA, USA), was used to prepare all BPA solutions for the adsorption/desorption tests.

### 2.2. Adsorption of Bisphenol A on TiO_2_ Nanoparticles

TiO_2_ nanopowder was weighed for addition to individual BPA solutions. These mixtures were homogenized by probe-sonication for 5–10 min. Dispersed TiO_2_ nanoparticle concentrations (≥50 µg/mL) were monitored using an ultraviolet (UV)–visible spectrophotometer (Thermo Scientific GENESYS 10S, Waltham, MA, USA). Wavelength scans of the sonicated TiO_2_ nanoparticle suspensions (200 to 700 nm) gave the typical spectrum with a peak at about 329 nm. The sample pH before and after adsorption were similar and close to neutral. The abundances of a target MS peak for BPA were measured before and after 3 h of mixing with TiO_2_ nanoparticles, either in the dark or in the room light under continuous magnetic stirring at 300 rpm. A *p* value <0.05 was considered statistically significant in the difference between the initial abundance (A_initial_) and experimental abundance (A_exp_) to validate that BPA adsorption occurred.

### 2.3. Desorption of Bisphenol A from TiO_2_ Nanoparticles

**Figure 1:**
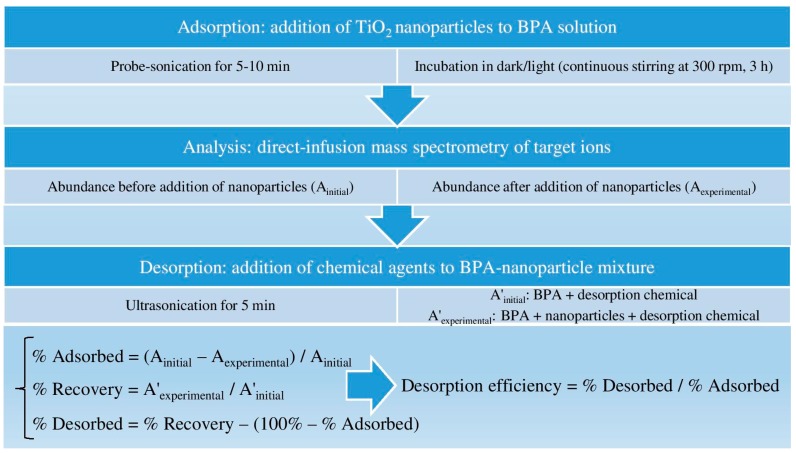
Summarizing scheme of the methods (BPA: bisphenol A, TiO_2_: titania).

Ammonium acetate, formate, fluoride, and hydroxide were evaluated as chemical agents for their desorption capabilities at various concentrations that are commonly used in the mobile phase of LC-MS analysis. The samples were sonicated for 5 min following the addition of desorption agent. Screening of the BPA-TiO_2_ mixtures by direct-infusion MS analysis was conducted to determine any efficient desorption of BPA for various concentrations of TiO_2_ nanoparticles. A *p* value <0.05 was considered statistically significant in the difference between the adsorbed abundance and final abundance (after adding a desorption agent) to validate BPA desorption. The optimum desorption conditions were applied to a mixture of 5 µg/mL BPA and 10 µg/mL nanoparticles to investigate desorption efficiency for low concentration levels. The % recovered was determined by comparing the experimental abundance (A’_exp_) with the initial abundance (A’_initial_) in the presence of a desorption agent, and desorption efficiency was calculated as the ratio of percentage desorbed to percentage adsorbed. All adsorption and desorption experiments were carried out in triplicate, and an average of triplicate measurements with standard deviation (SD) was reported (mean ± SD, *n* = 3). A summarizing scheme of the methods is presented in Figure 1.

### 2.4. Instrumentation

Full scan mass spectra were acquired by a mass selective detector (MSD) Quad SL system (Agilent, Mississauga, ON, USA) with an electrospray ionization (ESI) source, using the optimized parameters presented in Table 1. Each spectrum was obtained by averaging 12 sequential scans. All mass-to-charge ratio (*m*/*z*) values recorded in the spectra were rounded off to one decimal place for easy reading.

### 2.5. Sample Introduction to Mass Spectrometry

Clogging of the ESI needle (capillary) was cautionary even though its inner diameter was not small compared with the 25 nm anatase nanoparticles studied in this work. Initially, 50 µg/mL of BPA was treated with 200 µg/mL of TiO_2_ nanoparticles (a mixture of rutile and anatase, size < 100 nm) to investigate whether or not the peak corresponding to BPA could be diminished due to surface adsorption by the nanoparticles. The sample was homogenized by sonication for 10 min and incubated for 3 h at room temperature under continuous magnetic stirring. It was subsequently passed through a 0.22 µm syringe filter prior to MS injection. The sample was directly infused through a syringe pump (Harvard Apparatus, Holliston, MA, USA) at a flow rate of 50 µL/min. Unfortunately, the ESI needle did clog after few times injections and in between infusions because of high nanoparticle concentration and possibility of time-dependent aggregation inside the needle. Low concentrations (50–100 µg/mL) of nanoparticles helped alleviate the problem as the MS detection limit was good enough for the quantification of a small BPA amount adsorbed. To resolve the issue, the needle was first sonicated in methanol for 10 min for unclogging. After 3 h of adsorption, the sample containing TiO_2_ nanoparticles (<25 nm size) was sonicated again for 5 min and injected by the syringe pump. The syringe pump was further equipped with a 0.22 µm syringe filter prior to sample injection. The procedure was applied to nanoparticle concentrations as high as 500 µg/mL and no clogging of the needle was observed. In between sample infusions, the syringe, needle, tubing, and ESI chamber were washed by a continuous flow of HPLC-grade methanol or methanol:water (1:1 *v*/*v*) at 100 µL/min for 10 min. Although injection of nanoparticles at low concentration levels (<100 µg/mL as commonly used in adsorption studies) did not cause clogging of the capillary, the proposed procedure would be recommended to protect the ESI capillary and chamber for direct infusion of nanoparticles at high concentration levels (>200 µg/mL). It is worthwhile to note that the ESI chamber and capillary may require periodic cleaning after several injections of samples containing nanoparticles.

## 3. Results and Discussion

### 3.1. Adsorption of Bisphenol A on Titania Nanoparticles in Dark and Room Light (Positive-mode Electrospray Ionization Analysis)

**Figure 2:**
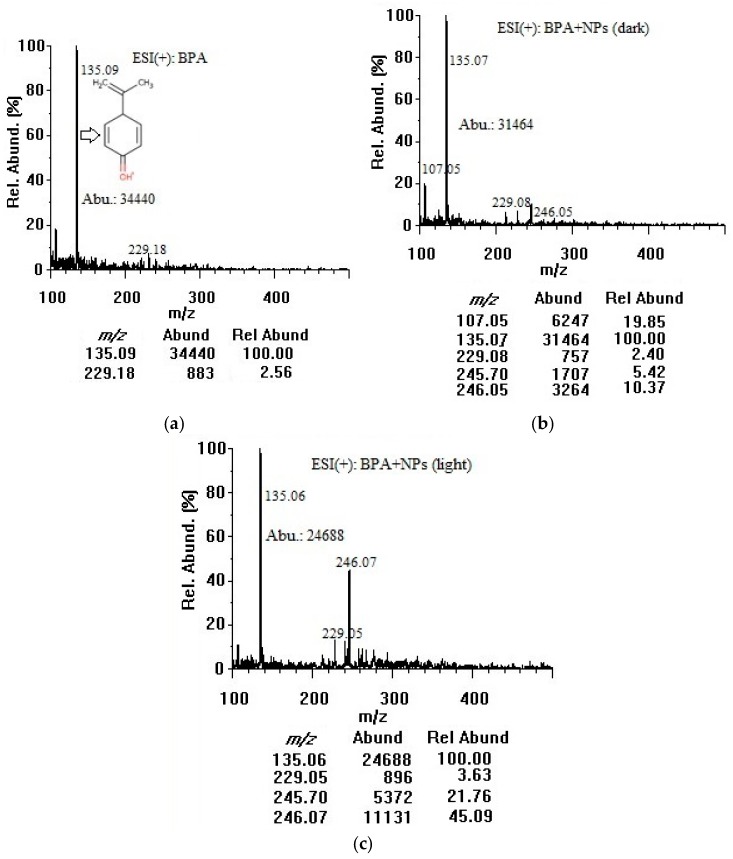
Positive-mode mass spectra of (**a**) BPA (50 µg/mL in water); (**b**) BPA (50 µg/mL) + TiO_2_ nanoparticles (100 µg/mL) after 3 h in dark; and (**c**) BPA (50 µg/mL) + TiO_2_ nanoparticles (100 µg/mL) after 3 h under room light (at an infusion flow rate of 100 µL/min). NPs: nanoparticles, Abu/Abund: abundance, Rel Abund: relative abundance.

Based on their exceptional physicochemical properties, TiO_2_ nanoparticles are most likely to adsorb organic contaminants in water [16]. In our study, BPA was chosen as an important compound to model the adsorption of organic contaminants onto colloidal TiO_2_ nanoparticles in water. The negative surface charge of the nanoparticles could be the main promoter of BPA adsorption. For our adsorption investigation, both positive and negative modes were employed and the ESI chamber-related parameters were optimized for better data interpretation. By the direct infusion of 50 µg/mL solutions, representative full scan ESI-MS spectra of BPA were acquired in the positive ion mode. The quality of MS spectra depended mainly on the TiO_2_ nanoparticle surface, where the molecular interactions would take place. Specifically, metal oxide–molecule charge transfer upon analyte adsorption was important. Figure 2 shows the full-scan mass spectra of 50 µg/mL BPA in the positive mode without (Figure 2a) and with 100 µg/mL TiO_2_ nanoparticles incubated for 3 h in the dark (Figure 2b) and under the room light (Figure 2c). A concentration of 50 µg/mL BPA was selected to primarily investigate the adsorption capability of TiO_2_ nanoparticles over a short period of time (3 h) and also to identify a potential chemical desorption agent. Such a concentration could produce pronounced differences in BPA signal intensities before and after treatment with nanoparticles. The optimum desorption conditions were then applied to 5 µg/mL BPA. As shown in Figure 2a, BPA could be ionized in the positive mode but the molecular ion abundance at *m*/*z* 229.1 corresponding to protonated BPA ([M + H]^+^) was low. The fragment ion at *m*/*z* 135.1 showed a higher abundance and was used as a target ion for all subsequent adsorption/desorption experiments. The *m*/*z* 107.1 peak was identified as [C_7_H_7_O]^+^, and the *m*/*z* 135.1 peak attributed to [C_9_H_11_O]^+^, as a result of cleavage of alkyl-phenyl bond and possible degradation of BPA [27,28,29]. The sodium adduct of the *m*/*z* 135.1 fragment showed a relative abundance of 34% at *m*/*z* 157.1 after addition of 10 mM sodium formate (data not shown). Although this adduct proved that the fragment was produced [30], the abundances of the fragment and its sodium adduct were so low during the injection of pure methanol indicating that the *m*/*z* 135.1 fragment did not originate from solvent contaminants or MS components. Furthermore, the abundance at *m*/*z* 135.1 increased linearly with increasing BPA concentration in a range of 5–100 µg/mL confirming that the fragment originated from BPA molecule. *Para*-isopropenylphenol ([M + H]^+^ at *m*/*z* 135.1) might also be produced as a result of photolytic [27], photocatalytic [28], and ultrasonic (sonochemical) degradation of BPA by hydroxyl radical generated from water and molecular oxygen dissociation [29]. The *m*/*z* 229.1 peak was assigned to the protonated molecular ion [M + H]^+^, and the *m*/*z* 245.7–246.1 peaks could be [M + NH_4_]^+^ [31]. No other adduct ions at detectable intensities were observed. In the dark, TiO_2_ nanoparticles bound ~9% of BPA when comparing the abundance of *m*/*z* 135.1 in the spectrum (b) with that of the spectrum (a). There might be some photocatalytic degradation of BPA even when the container was fully covered and kept in the dark during stirring because ultrasonic homogenization and sample filtration were conducted openly. Under room light, TiO_2_ nanoparticles bound and decomposed ~28% of BPA when comparing the abundance of *m*/*z* 135.1 in the spectrum (c) with that of the spectrum (a). The positive-mode mass spectra did not show any other additional peaks that could be assigned to photocatalytic degradation by-products of BPA [26]. The co-presence of nanoparticles could interfere with various steps in the mechanism of electrospray ionization. The bound BPA could be only partially desorbed and ionized, or the nanoparticles could suppress the ion abundance due to their interference (with aerosol formation, shrinkage to reach the Rayleigh limit, or evaporation of the water layer on a nanoparticle surface during drying/desolvation of BPA) to produce less ions in the gas phase for MS detection [31].

### 3.2. Adsorption of Bisphenol A on Titania Nanoparticles in Dark and Room Light (Negative-mode Electrospray Ionization Analysis)

**Figure 3:**
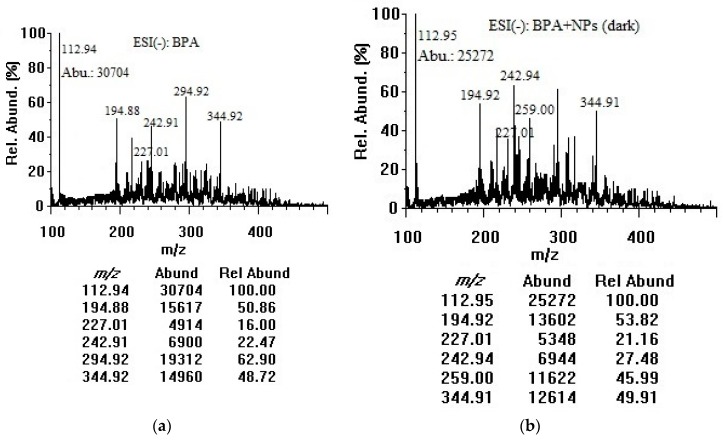

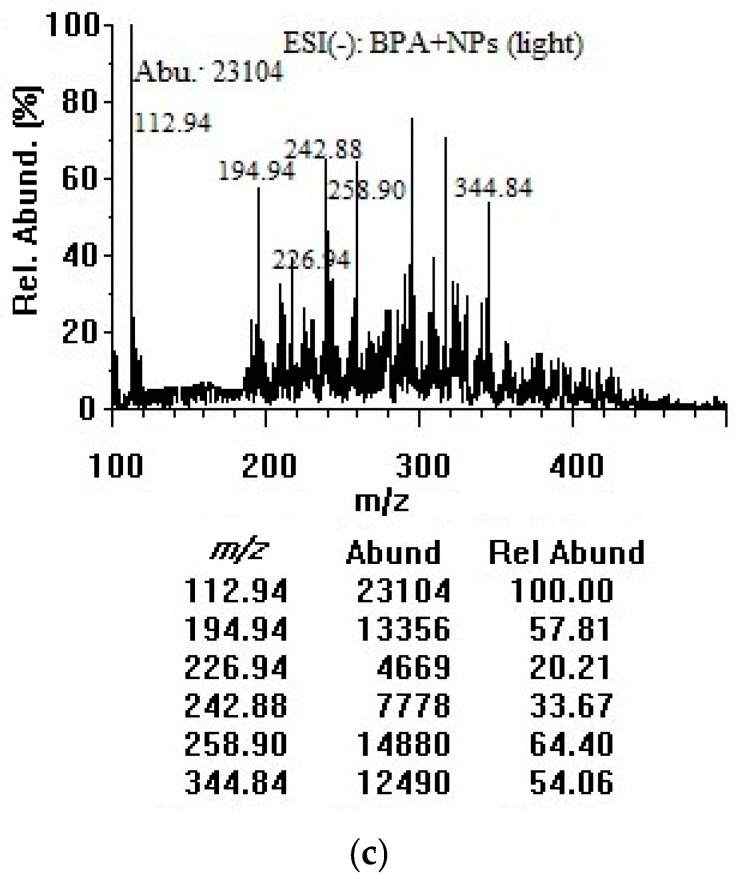
Negative-mode mass spectra of (**a**) BPA (50 µg/mL in water); (**b**) BPA (50 µg/mL) + TiO_2_ nanoparticles (100 µg/mL) after 3 h in dark; and (**c**) BPA (50 µg/mL) + TiO_2_ nanoparticles (100 µg/mL) after 3 h under room light. NPs: nanoparticles, Abu/Abund: abundance, Rel Abund: relative abundance.

BPA contains a hydrogen atom at the tertiary carbon atom in the α-position of each benzene ring and a hydroxyl group, enabling mass spectrometric detection of the molecular and deprotonated ions in the negative mode. Similar concentrations and experimental conditions were applied to acquire mass spectra in the negative mode. As illustrated in Figure 3, the *m*/*z* 113.0 peak was attributed to [M–2H]^2−^, the *m*/*z* 227.0 peak was assigned to the deprotonated molecular ion [M–H]^−^, and the *m*/*z* 294.9 could be for dichlorobisphenol A [M–H]^−^ [32]. The *m*/*z* 194.9 and 344.9 peaks could be for bisphenol AP and bisphenol P ([M–H–C_6_H_6_O]^−^ and [M–H]^−^) respectively, as the impurities [33]. There was a slight possibility that the *m*/*z* 194.9, *m*/*z* 294.9, and *m*/*z* 344.9 peaks arose from solvent contaminants or impurities. Deprotonated BPA in the sample solution could be strongly favored by the assistance of the hemiacetal oxygen lone pair, to produce singly and doubly deprotonated BPA in the ESI chamber. The peak at *m*/*z* 113.0 exhibited the highest abundances for BPA solutions without and with TiO_2_ nanoparticles in the dark and under room light as a result of loss of both acidic protons (double deprotonation of both hydroxyl groups) during the electrospray ionization process [34]. It was therefore used as the target ion in the negative mode. To determine if there were changes after mixing with TiO_2_ nanoparticles, one concentration of BPA solution (50 µg/mL) was combined with TiO_2_ nanoparticles (100 µg/mL). A decrease in the abundances at *m*/*z* 113.0 could be due to the adsorption/decomposition of BPA on the TiO_2_ nanoparticle surface. The peaks at *m*/*z* 242.9 and 259.0 could be attributed to [C_15_H_16_O_3_–H]^−^ and [C_15_H_16_O_4_–H]^−^ photocatalytic by-products [26].

### 3.3. Application of Mass Spectrometry Modifiers as Chemical Desorption Agents

As the ESI is a soft ionization method, it would be important to increase the sensitivity toward the target ions (for enhanced monitoring sensitivity) by applying buffers/modifiers or changing the chamber parameters. Our preliminary effort with 0.05% (*v*/*v*) formic acid did not enhance the ionization efficiency in the negative mode that much (only 15%–20%) compared to the positive mode but it promoted the photodegradation (sample pH of 4.3) for *m*/*z* 242.9 and 259.0 (data not shown herein). The electrospray ionization parameters of the mass spectrometer were adjusted to obtain the highest possible abundance of target ions, as summarized in Table 1. After ensuring that the sensitivity was sufficient to monitor target peaks amongst other background peaks nearby, we started to conduct adsorption experiments in the dark. Considering how simple the positive-mode spectra obtained above were, our desorption experiments was carried out in the positive mode. Although a good desorption agent for TiO_2_ nanoparticles could simply be Na_2_HPO_4_ (10–20 mM), phosphate as a non-volatile buffer ion may not be advisable for ESI. The use of stabilizing agents such as surfactants could influence desorption of BPA (and other emerging contaminants), but it could suppress electrospray ionization and mess up the mass spectrum badly.

**Figure 4:**
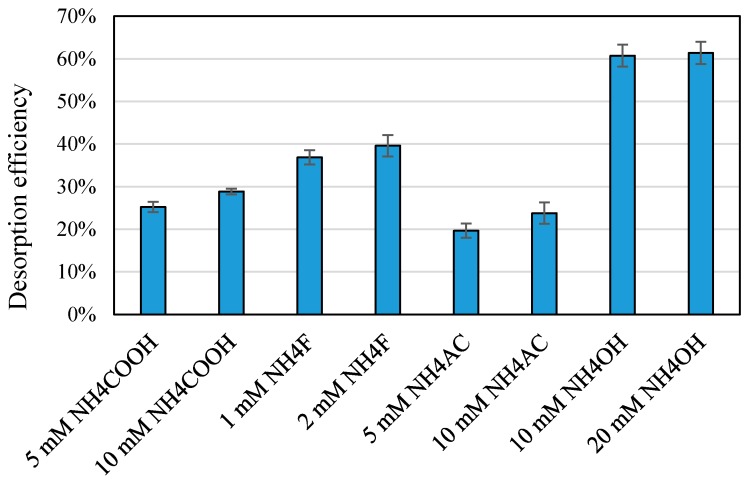
Effect of type and concentration of desorption agent on desorption efficiency for a mixture of 50 µg/mL BPA and 200 µg/mL TiO_2_ nanoparticles. Error bars represent standard deviations (*n* = 3).

Rather, we focused on other buffers/modifiers that could both enhance the ionization and might promote desorption-like ammonium acetate, ammonium fluoride, ammonium formate, and ammonium hydroxide. For instance, by changing the pH from the optimal range for maximized adsorption, desorption agent could modify the charge properties of BPA and TiO_2_ nanoparticles to enhance desorption. Figure 4 shows the effect of various concentration levels of desorption agents on desorption efficiency for a mixture of 50 µg/mL BPA and 200 µg/mL TiO_2_ nanoparticles. While the sensitivity (ionization efficiency) was observed to be higher in the presence of 1–2 mM ammonium fluoride than that in the presence of ammonium hydroxide (by comparing the abundances of the target adduct ion (*m*/*z* 135.1) in the positive mode before and after addition of ammonium fluoride), since the sample pH after adding ammonium fluoride to the mixture of BPA and TiO_2_ (between 6.7 and 6.9) was still close to the neutral range, desorption was not promoted efficiently. On the other hand, as observed for ammonium acetate and ammonium formate at concentration levels of 5 and 10 mM, while the sample pH was slightly above neutral range (pH between 7.1 and 7.4), the ionization efficiency in the ESI chamber was lower than ammonium fluoride. Furthermore, it has been reported that when ammonium acetate is introduced to the ESI chamber in positive ion mode, it could probably undergo acidification, resulting in a pH drop down to pH 4.7 in the ESI plume [35]. Thus, it could be deduced that both sample pH and ionization efficiency can influence desorption efficiency. In other words, modifiers like ammonium fluoride may increase desorption of BPA from the nanoparticle surface by enhancing the ionization of BPA and producing the ions more efficiently. As described earlier, electrospray ionization might contribute to partial desorption and ionization of the bound BPA molecules. The chemical desorption agents could enhance the ionization efficiencies of all free BPA, resulting in an increase of the calculated desorption efficiency. However, based on the results of adding 10 and 20 mM ammonium hydroxide (with the sample pH in the range of between 9.2 and 9.6), the sample pH had a more pronounced effect. As can be seen in Figure 4, while desorption efficiency increased by increasing the concentration of other desorption agents (as a result of enhanced ionization in the ESI chamber), the results for ammonium hydroxide were not significantly different since the sample pH was not varied markedly and the ionization efficiency was not also significantly affected (as expected for ammonium hydroxide in the positive mode). It should be noted that the chemical desorption agents used in this study did not react with BPA to produce new toxic chemicals under the experimental conditions applied. A comparison between full-scan mass spectra of BPA (without and with TiO_2_ nanoparticles) before and after addition of desorption chemicals (such as ammonium formate and ammonium fluoride) did not show extra peaks related to any new reaction products in both positive and negative polarities.

### 3.4. Ammonium Hydroxide as a Desorption Agent for Various Nanoparticle Concentrations

**Figure 5:**
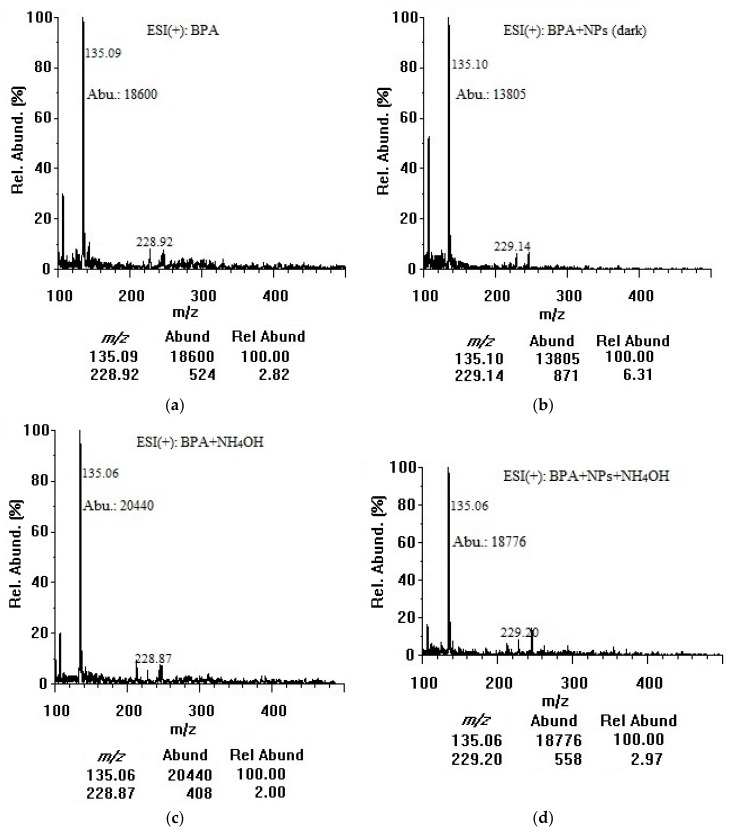
Positive-mode mass spectra of (**a**) BPA (50 µg/mL in water); (**b**) BPA (50 µg/mL) + TiO_2_ nanoparticles (100 µg/mL) after 3 h in dark; (**c**) BPA (50 µg/mL in water) + NH_4_OH (10 mM); and (**d**) BPA (50 µg/mL) + TiO_2_ nanoparticles (100 µg/mL) after 3 h in dark + NH_4_OH (10 mM) (at an infusion flow rate of 50 µL/min). NPs: nanoparticles, Abu/Abund: abundance, Rel Abund: relative abundance.

Figure 5 shows the results from desorption experiments in the dark for 100 µg/mL TiO_2_ nanoparticles. Firstly, a significant decrease in the abundance of target fragment ion (*m*/*z* 135.1) after 3 h in the presence of TiO_2_ nanoparticles indicated that BPA adsorption proceeded in the dark. Secondly, after adding 10 mM NH_4_OH, the pH reached 9.2 at which both TiO_2_ nanoparticles and BPA would be negatively charged (based on the isoelectric point of ≤7.5 for TiO_2_ nanoparticles and the pK_a_ of 9.73 for BPA) [36]. The samples were sonicated for 5 min following the addition of NH_4_OH. It should be noted that the ESI needle (capillary) and chamber were washed at the end of each experiment with 1:1 (*v*/*v*) methanol:water using the syringe pump at a flow rate of 100 µL/min to avoid harm due to alkaline conditions. While ammonium hydroxide did not enhance the sensitivity toward BPA (as expected for the positive mode), desorption was quite satisfactory by comparing the control (BPA with 10 mM NH_4_OH) with the sample (mixture of BPA and TiO_2_ nanoparticles) after the addition of NH_4_OH. Using these four abundance results, as summarized in Table 2, the percentage recovery was determined to be 92 ± 1% for 100 µg/mL TiO_2_ nanoparticles and 87 ± 1% for 200 µg/mL nanoparticles. The corresponding desorption efficiency was calculated to be 69 ± 1% and 61 ± 1%, respectively. This small decrease of desorption efficiency with increasing TiO_2_ nanoparticle concentration may be ascribed to a larger number of strong sites available for BPA binding. Importantly, in its present definition, desorption efficiency can serve as a marking scale for comparison of the four agents with others to be evaluated in future studies. These percentage calculations were also applied to plot the effects of nanoparticle concentration, and type/concentration of desorption agent (as reported in Figure 4).

**Figure 6:**
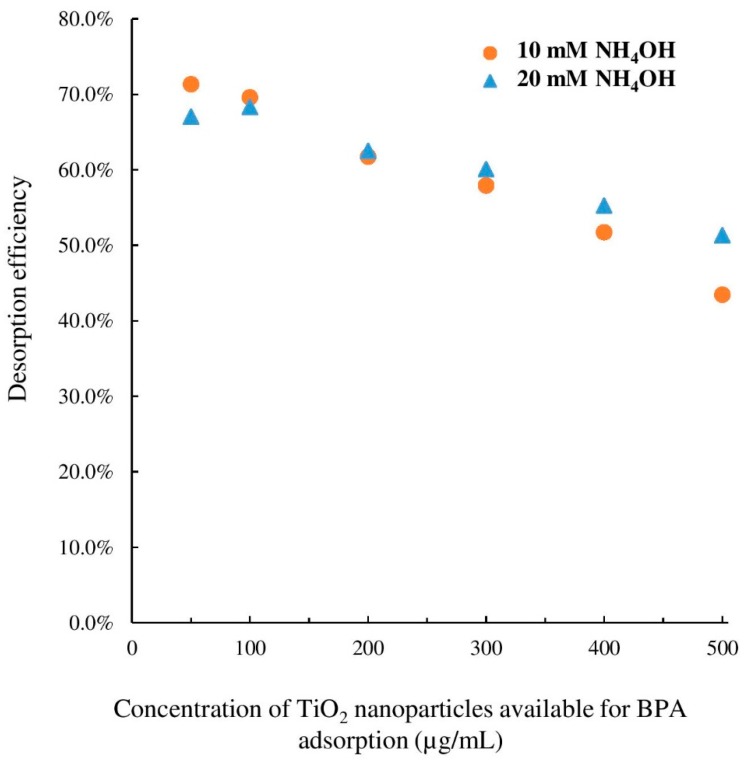
Effect of NH_4_OH concentration on BPA desorption from TiO_2_ nanoparticles. The results are the averages of triplicate measurements. Error bars were not shown for simplicity (relative standard deviation (RSD) between 3.9% and 8.4%).

As indicated in Table 2, desorption efficiency was significantly lower for very high concentration levels of TiO_2_ nanoparticles. The higher amount of ammonium hydroxide (20 mM) did not change desorption efficiency for 200 µg/mL TiO_2_ nanoparticles (as shown in Figure 4). However, as illustrated in Figure 6, desorption of BPA was significantly improved for 400 and 500 µg/mL TiO_2_ nanoparticles suggesting that a small variation of the sample pH under alkaline conditions can promote desorption of largely adsorbed percentage of BPA. However, since the results of lower concentration levels were not significantly different, 10 mM ammonium hydroxide was selected as the optimum level. This recommended value would avoid further potential harm to the MS parts due to highly alkali environment. Furthermore, such high concentration levels of TiO_2_ nanoparticles were solely used for the purpose of the evaluation of desorption efficiency in the ESI chamber by a desorption agent. As such, the optimum desorption agent and concentration was then applied to desorption of adsorbed BPA from TiO_2_ nanoparticles in a mixture of 5 µg/mL BPA and 10 µg/mL TiO_2_ nanoparticles. The exact adsorption and desorption protocols were followed. According to the calculation presented in Table 2, the percentage adsorbed, recovered, and desorbed were determined to be 27 ± 1%, 92 ± 1%, and 19 ± 1%, yielding a desorption efficiency of 72 ± 1%. It could be deduced that without a desorption mechanism, mass spectrometry was unable to accurately determine the concentration of BPA co-existing with TiO_2_ nanoparticles due to binding of BPA molecules onto the nanoparticle surface. On the other hand, by applying 10 mM ammonium hydroxide as an MS-friendly desorption chemical, both the free and bound forms of BPA could be determined with enhanced sensitivity and improved accuracy.

BPA fragment peak intensities were next examined to study the effect of photocatalysis on desorption. A meticulous inspection of these mass spectra allowed for the identification of by-products arising from the photodegradation of BPA. Ions at *m*/*z* 242.9 and 259.0 were clearly observed in the negative mode and assigned to the addition of hydroxyl groups producing [C_15_H_16_O_3_–H]^−^ and [C_15_H_16_O_4_–H]^−^ photocatalytic by-products (see Figure 3) [30]. However, these results were insufficient to explain the interactions between BPA and TiO_2_ nanoparticles. Based on the present experimental and previously reported theoretical results, we propose a new mechanistic hypothesis on desorption of BPA that may start from C1–OH protonation. When the easy proton shift among the BPA OH groups leads to the C1–OH protonated isomer, its facile dehydration triggers the BPA desorption reaction. Protonated BPA is not stable and promptly loses a water molecule giving rise to the dehydrated ion(s) at *m*/*z* 211.1 (and 193.1), although very low abundances (~3%–5% of the target adduct ion (*m*/*z* 135.1)) for those mass-to-charge ratios were observed since they were ultimately converted to [C_9_H_11_O]^+^ with an *m*/*z* of 135.1.

## 4. Conclusions

In this study, we developed a method for desorbing BPA from TiO_2_ nanoparticles by using carefully selected chemical agents. These desorption agents are suitable for the acquisition of mass spectra with minimal spectral interference from contaminants, which enables unambiguous identification of photocatalytic product ions. Based on MS analyses, ammonium hydroxide at concentration levels of 10 and 20 mM demonstrated the efficient desorption of BPA from TiO_2_ nanoparticles at various concentrations, as a result of its effects on the sample pH and electrospray ionization efficiency. It should be noted that the aim of this study was not to quantify the amount of BPA adsorbed on the surface of TiO_2_ nanoparticles. Since the nanoparticles were not separated from free BPA in the sample solutions prior to MS analysis, both free BPA and adsorbed BPA were introduced to the ESI chamber. As the adsorbed BPA could be partially desorbed and ionized on the nanoparticle surface, it would contribute to the observed signal intensity of target ions. Our results showed that ESI was unable to completely desorb/ionize the adsorbed BPA. To determine the percentage of adsorbed BPA that was eventually desorbed and ionized during electrospray ionization, or to investigate whether or not the process reached equilibrium in the ESI chamber, one would need to compare between the target ion abundance results for samples treated with TiO_2_ nanoparticles, with and without prior separation of nanoparticles from free BPA. Overall, this study demonstrated a simple, fast, and cost-effective method by which BPA could be detected in the presence of nanoparticles with enhanced accuracy. The method could be applied to real sample analysis where neither separation of nanoparticles from trace contaminants, nor discrimination between free and adsorbed species, would be feasible particularly when analyte preconcentration is required.

While the role of acetate, fluoride, formate, and hydroxide anions in combination with the ammonium cation in enabling desorption of BPA molecules from TiO_2_ nanoparticles is now established, only advanced analysis by MS/MS can provide characterization to identify the unknown fragment ions. Any ionic species derived from desorption reaction of protonated BPA can be structurally characterized by their fragmentation patterns. New knowledge of the ion structures and determination of their dissociation energy barriers may help validate the reaction mechanisms postulated by previous theoretical calculations. The formation of by-products could interfere with the adsorption study of BPA. While the role of desorption agents in the enhancement of ESI efficiency and pH adjustment (which would affect the nanoparticle surface charge and the ionic form of the adsorbed molecule depending on its pK_a_) may not be altered significantly, degradation of BPA could reduce the number of BPA molecules available for adsorption on the nanoparticle surface. To address this concern, light and ultrasonication (in terms of time and intensity) along with other experimental conditions need to be carefully controlled throughout the experiments to limit the formation of degradation by-products. In this regard, mass spectrometry could be highly advantageous as it would enable detecting various ions originating from BPA and its by-products, and it could monitor the changes of their abundances in the absence/presence of nanoparticles and chemical desorption agents under identical experimental conditions. The four desorption agents will be further tested for BPA desorption from three other most widely used metal oxide nanoparticles, namely ceria (CeO_2)_, copper(II) oxide (CuO) and zinc oxide (ZnO). Further studies would have to elucidate the optimum desorption conditions that may vary because of the characteristic/unique adsorption behavior and surface charge properties of these other nanoparticles. Their similarity and differences are not straightforwardly predictable.

## Figures and Tables

**Table 1:** Optimized source parameters for electrospray ionization (ESI) chamber.

Parameter	Value
Voltage (polarity)	−3.5 kV (negative) or +4.0 kV (positive)
Nebulizer gas (N_2_) pressure	15 psi
Drying gas flow rate	7.0 L/min
Drying gas temperature	300 °C
Mass-to-charge scan range	*m*/*z* 100–500

**Table 2:** Abundances of target fragment ion (*m*/*z* 135.1) in BPA sample solutions after treatment with 10 mM NH_4_OH.

TiO_2_ Concentration (µg/mL) Available for BPA Adsorption	Abundance of *m*/*z* 135.1 Peak (arb. Unit)		% Adsorbed = (A_initial_ − A_exp_)/A_initial_	% Recovery = A’_exp_/A’_initial_	% Desorbed = % Recovery-(100% − % Adsorbed)	Desorption Efficiency = % Desorbed/% Adsorbed
	A before Adding 10 mM NH_4_OH	A’ after Adding 10 mM NH_4_OH	before Adding 10 mM NH_4_OH	after Adding 10 mM NH_4_OH	by Adding 10 mM NH_4_OH	of 10 mM NH_4_OH
0	18,600	20,400	0.0%	100.0%	-	-
50	15,100	19,300	18.8%	94.6%	13.4%	71.3%
100	13,800	18,800	25.8%	92.2%	18.0%	69.6%
200	12,400	17,800	33.3%	86.9%	20.1%	60.6%
300	12,100	17,400	34.9%	85.3%	20.2%	57.9%
400	11,800	16,800	36.6%	82.4%	18.9%	51.7%
500	10,700	15,500	42.5%	76.0%	18.5%	43.4%

## References

[1] Adoamnei E., Mendiola J., Vela-Soria F., Fernández F., Olea N., Jørgensen N., Swan S.H., Torres-Cantero A.M. (2018). Urinary bisphenol A concentrations are associated with reproductive parameters in young men. Environ. Res..

[2] Cavanagh J.A.E., Trought K., Mitchell C., Northcott G., Tremblay L.A. (2018). Assessment of endocrine disruption and oxidative potential of bisphenol-A, triclosan, nonylphenol, diethylhexyl phthalate, galaxolide, and carbamazepine, common contaminants of municipal biosolids. Toxicol. In Vitro.

[3] Eladak S., Moison D., Guerquin M.J., Matilionyte G., Kilcoyne K., N’Tumba-Byn T., Messiaen S., Deceuninck Y., Pozzi-Gaudin S., Benachi A. (2018). Effects of environmental bisphenol A exposures on germ cell development and Leydig cell function in the human fetal testis. PLoS ONE.

[4] Galloway T.S., Baglin N., Lee B.P., Kocur A.L., Shepherd M.H., Steele A.M., BPA Schools Study Consortium, Harries L.W. (2018). An engaged research study to assess the effect of a ‘real-world’ dietary intervention on urinary bisphenol A (BPA) levels in teenagers. BMJ Open.

[5] Chen D., Kannan K., Tan H., Zheng Z., Feng Y.L., Wu Y., Widelka M. (2016). Bisphenol analogues other than BPA: Environmental occurrence, human exposure, and toxicity—A review. Environ. Sci. Technol..

[6] Chang H., Shen X., Shao B., Wu F. (2018). Sensitive analysis of steroid estrogens and bisphenol A in small volumes of water using isotope-dilution ultra-performance liquid chromatography-tandem mass spectrometry. Environ. Pollut..

[7] Li Y., Perera L., Coons L.A., Burns K.A., Ramsey J.T., Pelch K.E., Houtman R., van Beuningen R., Teng C.T., Korach K.S. (2018). Differential in vitro biological action, coregulator interactions, and molecular dynamic analysis of bisphenol A (BPA), BPAF, and BPS ligand–ERα complexes. Environ. Health Perspect..

[8] García-Córcoles M.T., Cipa M., Rodríguez-Gómez R., Rivas A., Olea-Serrano F., Vílchez J.L., Zafra-Gómez A. (2018). Determination of bisphenols with estrogenic activity in plastic packaged baby food samples using solid-liquid extraction and clean-up with dispersive sorbents followed by gas chromatography tandem mass spectrometry analysis. Talanta.

[9] Caballero-Casero N., Lunar L., Rubio S. (2016). Analytical methods for the determination of mixtures of bisphenols and derivatives in human and environmental exposure sources and biological fluids. A review. Anal. Chim. Acta.

[10] Acosta R., Nabarlatz D., Sánchez-Sánchez A., Jagiello J., Gadonneix P., Celzard A., Fierro V. (2018). Adsorption of bisphenol A on KOH-activated tire pyrolysis char. J. Environ. Chem. Eng..

[11] Zhu H., Li Z., Yang J. (2018). A novel composite hydrogel for adsorption and photocatalytic degradation of bisphenol A by visible light irradiation. Chem. Eng. J..

[12] Bhadra B.N., Lee J.K., Cho C.W., Jhung S.H. (2018). Remarkably efficient adsorbent for the removal of bisphenol A from water: Bio-MOF-1-derived porous carbon. Chem. Eng. J..

[13] Li X., Zhou M., Jia J., Ma J., Jia Q. (2018). Design of a hyper-crosslinked β-cyclodextrin porous polymer for highly efficient removal toward bisphenol A from water. Sep. Purif. Technol..

[14] Choong C.E., Ibrahim S., Yoon Y., Jang M. (2018). Removal of lead and bisphenol a using magnesium silicate impregnated palm-shell waste powdered activated carbon: Comparative studies on single and binary pollutant adsorption. Ecotoxicol. Environ. Saf..

[15] Kusvuran E., Yildirim D. (2013). Degradation of bisphenol A by ozonation and determination of degradation intermediates by gas chromatography-mass spectrometry and liquid chromatography-mass spectrometry. Chem. Eng. J..

[16] Yan J., Lin B., Hu C., Zhang H., Lin Z., Xi Z. (2014). The combined toxicological effects of titanium dioxide nanoparticles and bisphenol A on zebrafish embryos. Nanoscale Res. Lett..

[17] Rehse S., Kloas W., Zarfl C. (2018). Microplastics reduce short-term effects of environmental contaminants. Part I: Effects of bisphenol a on freshwater zooplankton are lower in presence of polyamide particles. Int. J. Environ. Res. Public Health.

[18] Verdier T., Bertron A., Erable B., Roques C. (2018). Bacterial biofilm characterization and microscopic evaluation of the antibacterial properties of a photocatalytic coating protecting building material. Coatings.

[19] Dhanasekar M., Jenefer V., Nambiar R.B., Babu S.G., Selvam S.P., Neppolian B., Bhat S.V. (2018). Ambient light antimicrobial activity of reduced graphene oxide supported metal doped TiO_2_ nanoparticles and their PVA based polymer nanocomposite films. Mater. Res. Bull..

[20] Hosseini-Zori M. (2018). Co-doped TiO_2_ nanostructures as a strong antibacterial agent and self-cleaning cover: Synthesis, characterization and investigation of photocatalytic activity under UV irradiation. J. Photochem. Photobiol. B Biol..

[21] Žerjav G., Kaplan R., Pintar A. (2018). Catalytic wet air oxidation of bisphenol A aqueous solution in trickle-bed reactor over single TiO_2_ polymorphs and their mixtures. J. Environ. Chem. Eng..

[22] Žerjav G., Djinović P., Pintar A. (2018). TiO_2_-Bi_2_O_3_/(BiO)_2_CO_3_-reduced graphene oxide composite as an effective visible light photocatalyst for degradation of aqueous bisphenol A solutions. Catal. Today.

[23] Dos Santos D.M., Williams M., Kookana R., de Marchi M.R.R. (2018). Predicting bioaccessibility of contaminants of emerging concern in marine sediments using chemical methods. J. Soil Sediment.

[24] Zheng D., Wang N., Wang X., Tang Y., Zhu L., Huang Z., Tang H., Shi Y., Wu Y., Zhang M. (2012). Effects of the interaction of TiO_2_ nanoparticles with bisphenol A on their physicochemical properties and in vitro toxicity. J. Hazard. Mater..

[25] Deng R., Lin D., Zhu L., Majumdar S., White J.C., Gardea-Torresdey J.L., Xing B. (2017). Nanoparticle interactions with co-existing contaminants: Joint toxicity, bioaccumulation and risk. Nanotoxicology.

[26] Silva J.C.C., Reis Teodoro J.A., de Cássia Franco Afonso R.J., Aquino S.F., Augusti R. (2014). Photodegradation of bisphenol A in aqueous medium: Monitoring and identification of by-products by liquid chromatography coupled to high-resolution mass spectrometry. Rapid Commun. Mass Spectrom..

[27] Chen P.J., Linden K.G., Hinton D.E., Kashiwada S., Rosenfeldt E.J., Kullman S.W. (2006). Biological assessment of bisphenol A degradation in water following direct photolysis and UV advanced oxidation. Chemosphere.

[28] Guo C., Ge M., Liu L., Gao G., Feng Y., Wang Y. (2010). Directed synthesis of mesoporous TiO_2_ microspheres: Catalysts and their photocatalysis for bisphenol A degradation. Environ. Sci. Technol..

[29] Torres R.A., Pétrier C., Combet E., Carrier M., Pulgarin C. (2008). Ultrasonic cavitation applied to the treatment of bisphenol A. Effect of sonochemical parameters and analysis of BPA by-products. Ultrason. Sonochem..

[30] Aznar M., Alfaro P., Nerin C., Kabir A., Furton K.G. (2016). Fabric phase sorptive extraction: An innovative sample preparation approach applied to the analysis of specific migration from food packaging. Anal. Chim. Acta.

[31] Konermann L., Ahadi E., Rodriguez A.D., Vahidi S. (2013). Unraveling the mechanism of electrospray ionization. Anal. Chem..

[32] Gallart-Ayala H., Moyano E., Galceran M.T. (2007). Liquid chromatography/multi-stage mass spectrometry of bisphenol A and its halogenated derivatives. Rapid Commun. Mass Spectrom..

[33] Zhao H., Xiang L., Li J., Yang Z., Fang J., Zhao C., Xu S., Cai Z. (2016). Investigation on fragmentation pathways of bisphenols by using electrospray ionization Orbitrap mass spectrometry. Rapid Commun. Mass. Spectrom..

[34] Maragou N.C., Lampi E.N., Thomaidis N.S., Koupparis M.A. (2006). Determination of bisphenol A in milk by solid phase extraction and liquid chromatography-mass spectrometry. J. Chromatogr. A.

[35] Konermann L. (2017). Addressing a common misconception: Ammonium acetate as neutral pH “buffer” for native electrospray mass spectrometry. J. Am. Soc. Mass Spectrom..

[36] Fernández-Nieves A., de las Nieves F.J., Richter C., Koper G.J.M., Bedeaux D., Cavaco C., Sager W.F.C. (1998). Point of zero charge estimation for a TiO_2_/water interface. Trends in Colloid and Interface Science XII. Progress in Colloid and Polymer Science.

